# Inability to Walk Predicts Death among Adult Patients in Hospitals in Malawi

**DOI:** 10.1155/2019/6586891

**Published:** 2019-07-07

**Authors:** Raphael Kazidule Kayambankadzanja, Carl Otto Schell, Grace Nsanjama, Isaac Mbingwani, Samson Kwazizira Mndolo, Jamie Rylance, Tim Baker

**Affiliations:** ^1^University of Malawi, College of Medicine, Blantyre, Malawi; ^2^Department of Anaesthesia and Intensive Care, Queen Elizabeth Central Hospital, Blantyre, Malawi; ^3^Global Health-Health Systems and Policy, Department of Public Health Sciences, Karolinska Institute, Stockholm, Sweden; ^4^Centre for Clinical Research Sörmland, Uppsala University, Uppsala, Sweden; ^5^Department of Internal Medicine, Nyköping Hospital, Sörmland Region, Nyköping, Sweden; ^6^Chiradzulu District Hospital, Malawi; ^7^Department of Clinical Sciences, Liverpool School of Tropical Medicine, Liverpool, UK; ^8^Malawi-Liverpool-Wellcome Trust Clinical Research Programme, Blantyre, Malawi

## Abstract

**Objective:**

Vital signs are often used in triage, but some may be difficult to assess in low-resource settings. A patient's ability to walk is a simple and rapid sign that requires no equipment or expertise. This study aimed to determine the predictive performance for death of an inability to walk among hospitalized Malawian adults and to compare its predictive value with the vital signs-based National Early Warning Score (NEWS).

**Methods:**

It is a prospective cohort study of adult in-patients on selected days in two hospitals in Malawi. Patients were asked to walk five steps with close observation and their vital signs were assessed. Sensitivities, specificities, and predictive values for in-patient death of an inability to walk were calculated and an inability to walk was compared with NEWS.

**Results:**

Four-hundred and forty-three of the 1094 participants (40.5%) were unable to walk independently. In this group, 70 (15.8 %) died in-hospital compared to 16 (2.5%) among those who could walk: OR 7.4 (95% CI 4.3-13.0 p<0.001). Inability to walk had a sensitivity for death of 81.4%, specificity of 63.0%, positive predictive value (PPV) of 15.8%, and negative predictive value (NPV) of 97.5%. NEWS>6 had sensitivity 70.9%, specificity 70.6%, PPV 17.1%, and NPV 96.6%. An inability to walk had a fair concordance with NEWS>6 (kappa 0.21).

**Conclusion:**

Inability to walk predicted mortality as well as NEWS among hospitalized adults in Malawi. Patients who were able to walk had a low risk of death. Walking ability could be considered an additional vital sign and may be useful for triage.

## 1. Introduction

Simple physiological signs are often used in hospitals as markers of illness severity [1, 2]. In emergency departments, triage systems routinely use vital signs such as blood pressure, heart rate, and respiratory rate to stratify patients into risk groups [3, 4]. In recent years, the need for ward-based triage has increasingly been recognized, leading to the development of Early Warning Scores such as the National Early Warning Score (NEWS) which has become widespread in the UK and other countries [5–7].

Many of the scoring systems use compound scores requiring a calculation of an overall score based on several components. This may be too time-consuming for efficient use in settings of low human resources, and time-pressured staff often miscalculate the scores leading to misclassification of patients [8, 9]. Moreover, the monitoring of vital signs such as blood pressure requires equipment and training for the health workers, which may be in short supply [10, 11]. A further limitation of warning scores is their predictive performance, which while in some settings has been found to be reasonable [12], was not accurate enough for clinical use in others [13, 14].

An ideal physiological sign of risk in a low-resource setting would be one that is simple and quick to assess, does not require equipment or special training for staff, and had a good performance for dividing patients into risk groups. A patient's ability to walk has been proposed as such a sign [15, 16] and has shown an association with mortality among medical admissions in Tanzania [17]. This study aimed to determine the predictive performance for death of an inability to walk among hospitalized adults in two hospitals in Malawi and to compare inability to walk with NEWS.

## 2. Methodology

### 2.1. Study Design

A prospective cohort study of adult in-patients recruited on single selected days with a primary outcome of in-hospital mortality.

### 2.2. Study Setting

The study was conducted in Queen Elizabeth Central Hospital (QECH) and Chiradzulu District Hospital (CDH). QECH is a national referral hospital located in Blantyre in the south of Malawi with a 1000-bed capacity, including 4 adult intensive care beds, serving an immediate catchment population of 1 million people. CDH is a district hospital with a catchment population of 250,000 and a 300-bed capacity (no intensive care). These two hospitals were chosen to provide settings of different resources, personnel, and epidemiology.

### 2.3. Study Population

All in-patients aged 18 and above in QECH and CDH on the selected days—twice in QECH in January 2017 and May 2018 and three times in CDH in November 2017, February 2018 and July 2018—were included. Exclusion criteria were refusal to participate, healthy prenatal pregnant women, women in active labour, and patients with a psychiatric disorder as the primary reason for admission.

### 2.4. Data Collection

A clinical evaluation of every patient was conducted by study nurses following training and using standardized methods and equipment. Patients were asked to stand up and walk five steps. Under close observation by a study nurse, each patient was coded as walking independently, walking with assistance, unable to walk, or refused to walk. Blood pressure was measured using Omron M2 digital blood pressure machines, oxygen saturations and heart rates were measured using Lifebox pulse oximeters, and axillary temperatures were taken using Ishnee IN 101A digital thermometers. Demographic and clinical information were extracted from the patients' files. Outcomes were collected by the ward clerks and the research team with a primary end-point of in-hospital mortality, censored at 30 days.

Ethical clearance was granted by the University of Malawi College of Medicine Research and Ethics Committee (COMREC P.08/16/2007).

### 2.5. Statistical Analysis

For the primary analyses, the patients' ability to walk was recategorised into two groups: those not able to walk independently (unable to walk, walking with assistance, or refuse to walk) and those able to walk independently. NEWS was scored between 0 and 20 according to the established system based on vital sign derangements [5]. A critical NEWS score was defined using the recognized cut-off of NEWS>6, and an alternative cut-off of NEWS>3 was also investigated. Patient characteristics were summarized using proportions, means, and ranges were appropriate. The predictive performance for death of inability to walk, NEWS>6, NEWS>3, and combined scores were analysed using logistic regression, sensitivity, specificity, and positive and negative predictive values. Area Under the Receiver Operating Characteristic Curve (AUROC) analysis was used for the semicontinuous NEWS score. The concordance of inability to walk with NEWS values was evaluated using kappa scores. Kappa scores assess how well two measures agree with each other and can have a value between 0 and 1 where poor <0.20; fair 0.21–0.40; moderate 0.41–0.60; good 0.61–0.80, and very good 0.81–1.0 [18]. A sensitivity analysis was done where we reclassified patients who were unable to walk due to physical impairment as “able to walk independently.” Data analysis was conducted using STATA (Release 15, StataCorp, College Station, TX).

## 3. Results

Of the 1135 eligible adults in the hospital on the study days, data on walking status or hospital outcomes were not collected for 41 (3.6%) and 1094 patients were included in the study from medicine, surgery, obstetrics and gynaecology, ophthalmology, and orthopaedics (Table 1 and supplementary Table 1). The participants mean age was 39 (range 18 to 98 years), 648 (59.2%) were female (Table 1), and demographics were similar at the two sites (Supplementary Table 2). Eighty-six patients died giving an in-hospital mortality rate of 7.9%: 9.1% in QECH and 3.4% in CDH.

Among the participants, 443 (40.5%) were unable to walk independently (220 were unable to walk, 214 could walk with assistance, and 9 refused to walk). Seventy of them died (15.8%) compared to 16 (2.5%) among those who could walk: OR 7.4 (95% CI 4.3-13.0 p<0.001). As a predictor of in-hospital mortality, inability to walk independently had a sensitivity of 81.4%, specificity of 63.0%, positive predictive value (PPV) of 15.8%, and negative predictive value (NPV) of 97.5%. In the sensitivity analysis, one hundred and thirteen were not able to walk independently due to a physical impairment and were classified as able to walk independently. As a predictor of in hospital mortality, an inability to walk that was not due to physical impairment (355 patients) had an OR 11.1 (CI 6.3-19.4 p<0.001) a sensitivity of 81.4%, and a specificity of 71.7% with a PPV of 19.7 % and an NPV of 97.8%.

Of the 357 patients with NEWS>6, 61 (17.1%) died in-hospital, compared to 25 (3.4%) of those with NEWS≤6: OR 7.2 (CI 3.6-9.5 p<0.001). For predicting in-hospital mortality, NEWS>6 had a sensitivity of 70.9% and specificity of 70.6% with a PPV of 17.1% and an NPV of 96.6%. The AUROC for NEWS was 0.76 (see Table 2).

Of the 930 patients with NEWS>3, 82 (8.8%) died in-hospital, compared to 2.4% of those with NEWS ≤3: OR 3.9 (CI 1.4-10.7 p=0.009). As a predictor of in hospital mortality, NEWS>3 had a sensitivity of 95.3% and specificity of 16.0% with a PPV of 8.8% and an NPV of 97.6%.

An inability to walk had a fair concordance with NEWS>6: kappa 0.21, 95% CI (0.16 - 0.27), and a poor concordance with NEWS>3: kappa 0.06, 95% CI (0.03 - 0.10).

Combining inability to walk with NEWS was done using “*and*” (both pathological signs necessary) and “*or*” (either pathological signs necessary) approaches. Among the 199 patients who were unable to walk* and* had NEWS>6, 53 (26.6%) died compared to 33 (3.7%) of those who were not positive for both pathological signs: OR 9.5 (CI 5.9-15.2 p<0.001). The sensitivity for in-hospital death of this “and” compound variable was 61.6% and specificity 85.5% with a PPV of 26.6% and an NPV of 96.3%.

Among the 601 patients who were unable to walk* or* had NEWS>6, 78 (13.0%) died compared to 8 (1.6%) of the 493 who had neither pathological sign: OR 9.0 (CI 4.3-19.0 p<0.001). The sensitivity for in-hospital death of this “or” compound variable was 90.7% and specificity 48.1% with a PPV of 13.0% and an NPV of 98.4%.

## 4. Discussion

We have found that an inability to walk was associated with death among hospitalized adults in Malawi. Inability to walk had as good a predictive performance as the compound vital signs-based NEWS score.

An inability to walk may be a marker of generalised deranged physiology and illness severity, or a reduced physiological reserve for coping with subsequent stresses and may include subtle signals not fully reflected by traditional vital signs or NEWS [15]. Our findings are similar to those from Tanzania where an inability to walk among adult patients at admission had increased odds of dying in hospital (OR 6.5) [17] as well as in a retrospective analysis of 7-day mortality from Uganda and Denmark (OR 11.8 and 6.7, respectively) [15].

Our finding that may be of most clinical utility is inability to walk has high negative predictive value – 97.5%. Only 2.5% of patients who were able to walk independently subsequently died in-hospital. The ease of assessing ability to walk and the low NPV could be used clinically for quickly “ruling-out” patients in need of urgent attention—a concept similar to the “walking wounded” in military situations, where injured soldiers who can still walk are triaged as a low-risk group to wait for medical assistance [19].

Concordance of an inability to walk with a high NEWS was low, implying that an inability to walk identified a different group of high-risk patients to those identified by NEWS and a potential for combining triage signs. Indeed, when combining inability to walk with NEWS, three risk groups could be identified: one-in-four (26.6%) of those with both pathological signs died in-hospital, one-in-seven (13.0%) of those with either an inability to walk or NEWS>6 died, and fewer than one-in-sixty (1.6%) of those who could both walk and NEWS<6 died in-hospital. When inability to walk was added to NEWS>6 using an “and” approach (both pathological signs necessary), the sensitivity for death dropped from 70.9% to 61.6% and specificity increased from 70.6% to 85.5%. When inability to walk was combined to NEWS>6 using an “or” approach (either pathological sign necessary), the sensitivity increased from 70.9% to 90.7% and specificity decreased from 70.6% to 48.1%.

Strength of our study is its use of a simple, pragmatic, and low-cost triage tool that does not require any equipment or expertise and would be possible in all resource levels. Our prospective study is unique in its focus on inpatients rather than patients at arrival to hospital—in-hospital triage and stratification of patients into risk groups is gathering interest, and this study is the first to look at such a simple triage criterion among an unselected patient population in a low-resource setting. While the methodology of collecting data from all hospital patients on selected days has such strengths, it also has limitations in that it does not capture the fluctuating nature of the conditions of patients or the effects of received therapies. Other limitations are that it was conducted in only two centres and data collection was done on only a few days.

Our study suggests that assessing individual patients' ability to walk may be a quick and easy tool for the stratification of patients into high- and low-risk groups. In settings where regular checks of vital signs are difficult or impossible, an ability to walk may have the potential to be a pragmatic alternative and could be part of essential emergency and critical care services [20]. In settings where vital signs can be checked, an additional assessment of the ability to walk may be useful for identifying patients at high risk who had low NEWS scores, or to find those at low-risk who may safely wait for treatment or be discharged. More work is required to explore the predictive value and optimal use of inability to walk and intervention studies should be conducted to assess the impact of introducing this simple assessment tool into clinical practice.

## 5. Conclusion

Inability to walk predicted mortality as well as NEWS among hospitalized adults in Malawi. Patients who were able to walk had a low risk of in-hospital death. Walking ability could be considered an additional vital sign and may be useful for triage.

## Figures and Tables

**Table 1:** Patient characteristics.

	N=1094

Female, n (%)	648 (59.2)

Age, mean years (range)	39 (18-98)

HIV positive, n/N^*∗*^ (%)	354/814 (43.5)

Diagnoses^*∗∗*^	
Post-delivery or post-abortion care	209 (19.1%)
Trauma	150 (13.7%)
Cancer	129 (11.8%)
Bacterial infection / sepsis	84 (7.7%)
Tuberculosis	83 (7.6%)
Non-communicable diseases	60 (5.5%)
Pneumonia	48 (4.4%)
Anaemia	44 (4.0%)
Meningitis	38 (3.5%)
Malaria	16 (1.5%)
Pre-eclampsia/eclampsia	13 (1.2%)
Other/unknown	220 (20.1%)

Specialty, n (%)	
Medicine^*∗∗∗*^	457 (41.8)
Surgery^*∗∗∗∗*^	285 (26.1)
Obstetrics and Gynaecology	281(25.7)
Ophthalmology	45 (4.1)
Orthopaedics	26 (2.4)

Length of hospital stay, median days (IQR)	
Before data collection	5(2-12)
After data collection^*∗∗∗∗∗*^	4(2-10)

^*∗*^ known HIV status N=814

^*∗∗*^ primary diagnosis at time of data collection

^*∗∗∗*^ including dermatology, tuberculosis and oncology.

^*∗∗∗∗*^ including ear-nose-throat surgery, neurosurgery and plastic surgery

^*∗∗∗∗∗*^ data censored at 30 days

**Table 2:** In-hospital mortality by walking ability and NEWS.

Variable		N (%)	Mortality n (%)	OR	95% CI	P value
All		1094	86 (7.8%)	-	-	-

Walking status	Able to walk independently	651 (59.5 %)	16 (2.5 %)	ref	ref	ref
	Unable to walk independently^*∗*^	443 (40.5 %)	70 (15.8 %)	7.4	4.3-13.0	<0.001
	Able to walk independently	651 (59.5 %)	16 (2.5 %)	ref	ref	ref
	Able to walk with assistance	214 (19.6%)	30 (14.0%)	6.5	3.5-12.1	<0.001
	Unable to walk	220 (20.1%)	39 (17.7%)	8.6	4.7-15.7	<0.001
	Refused to walk	9 (0.8%)	1 (11.1%)	5.0	0.6-42.0	0.142

NEWS	NEWS≤6	737(67.4%/	25 (3.4%)	ref	ref	ref
	NEWS>6	357(32.6%)	61 (17.1%)	5.9	3.6-9.5	<0.001
	NEWS≤3	164 (15.0%)	4 (2.4%)	ref	ref	ref
	NEWS>3	930 (85.0%)	82 (8.8%)	3.9	1.4-10.7	0.009

Combined	Able to walk and/or NEWS≤6	895 (81.8%)	33 (3.7%)	ref	ref	ref
	Unable to walk and NEWS>6	199 (18.2%)	53 (26.6%)	9.5	5.9-15.2	<0.001
	Able to walk and NEWS≤6	493 (45.1%)	8 (1.6%)	ref	ref	ref
	Unable to walk or NEWS>6	601 (54.9%)	78 (13.0%)	9.0	4.3-19.0	<0.001

^*∗*^ Variable generated by combining those able to walk with assistance, unable to walk and refused to walk

NEWS National Early Warning Score OR Odds Ratio

## Data Availability

The data used to support the findings of this study are available from the corresponding author upon request.

## Supplementary Materials

Supplementary Materials Supplementary table 1 Ability to walk and outcomes by specialty. Supplementary table 2. Patient characteristics by hospital.
