# Supplementary material for: Inability to Walk Predicts Death among Adult Patients in Hospitals in Malawi
Source: Emerg Med Int. 2019 Jul 7;2019:6586891. doi: 10.1155/2019/6586891 (PMC6642788; doi:10.1155/2019/6586891)
Supplement: Supplementary Materials — Supplementary table 1 Ability to walk and outcomes by specialty. Supplementary table 2. Patient characteristics by hospital. [file 6586891.f1.pdf]

Supplementary table 1 Ability to walk and outcomes by specialty

Specialty	All	Medicine	Surgery	Obstetrics and Gynaecology	Ophthalmology	Orthopedics
	1094	457(41.8%)	285(26.1%)	281 (25.7%)	45 (4.1%)	26 (2.4%)
Walking status<br>Able to walk Independently<br><br>Unable to walk independently	 651 (59.5%)<br><br>443 (40.5%)	 241 (52.7%)<br><br>216 (47.3%)	 127 (44.6%)<br><br>158 (55.4%)	 239 (85.1%)<br><br>42 (15.0%)	 40 (88.9%)<br><br>5 (11.1%)	 4 (15.4%)<br><br>22 (84.6%)
Number of in-hospital deaths n (%)	86 (7.9%)	66 (14.4%)	19 (6.7%)	1 (0.4%)	0	0
Odd ratio of death if unable to walk independently OR (95% CI)	7.4 (4.2-13.0) p<0.001	6.4 (3.3-12.3) p<0.001	4.7 (1.3-16.4) p=0.016	-	-	-

OR odds ratio CI confidence interval

Supplementary table 2. Patient characteristics by hospital

	Chiradzulu	QECH
	N=236	N=858
Female n (%)	163 (69.1)	485 (56.5)
Age, mean years(range)	39 (18-91)	39 (18-98)
HIV positive*, n (%)	73 (41)	281 (44.2)
Specialty, n (%)		
Medicine**	108 (45.8)	349 (40.7)
Surgery***	53 (22.5)	232 (27.0)
Obstetrics and Gynaecology	75 (31.8)	206 (24.0)
Ophthalmology	0 (0)	45 (5.2)
Orthopaedics	0 (0)	26 (3.0)
Length of hospital stay, median days (IQR)		
Before data collection	3.0 (1.0-6.0)	5.0(2.0-10.9)
After data collection****	3.0 (1.0-6.0)	5.0(2.0-10.8)
In hospital mortality	8 (3.4)	78 (9.1)
Walking status, n (%)		
Able to walking independently	157 (66.5)	494 (57.6)
Unable to walk independently*****	79 (33.5)	364 (42.0)*
Able to walk with assistance	33 (14.0)	181(21.1)
Unable to walk	41 (17.4)	179(20.9)
Refused to walk	5 (2.1)	4 (0.05)

* of those with known HIV status

[**] including dermatology, tuberculosis and oncology.
[***] including ear-nose-throat surgery, neurosurgery and plastic surgery
[****] data censored at 30 days
[*****] Variable generated by combining those able to walk with assistance, unable to walk and refused to walk
